# The effect of optimistic expectancies on attention bias: Neural and behavioral correlates

**DOI:** 10.1038/s41598-020-61440-1

**Published:** 2020-04-16

**Authors:** Laura Singh, Laurent Schüpbach, Dominik A. Moser, Roland Wiest, Erno J. Hermans, Tatjana Aue

**Affiliations:** 1 Department of Psychology, University of Bern, 3012 Bern, Switzerland; 2 Institute of Diagnostic and Interventional Neuroradiology, University Hospital, Inselspital, University of Bern, 3010 Bern, Switzerland; 3 Donders Institute for Brain, Cognition and Behaviour, Radboud University Medical Center, 6500 HB Nijmegen, The Netherlands

**Keywords:** Attention, Insula, Motivation, Reward

## Abstract

Optimism bias and positive attention bias are important features of healthy information processing. Recent findings suggest dynamic bidirectional optimism-attention interactions, but the underlying neural mechanisms remain to be identified. The current functional magnetic resonance imaging (fMRI) study, therefore, investigated the neural mechanisms underlying causal effects of optimistic expectancies on attention. We hypothesized that expectancies guide attention to confirmatory evidence in the environment, with enhanced salience and executive control network (SN/ECN) activity for unexpected information. Moreover, based on previous findings, we anticipated optimistic expectancies to more strongly impact attention and SN/ECN activity than pessimistic expectancies. Expectancies were induced with visual cues in 50 participants; subsequent attention to reward and punishment was assessed in a visual search task. As hypothesized, cues shortened reaction times to expected information, and unexpected information enhanced SN/ECN activity. Notably, these effects were stronger for optimistic than pessimistic expectancy cues. Our findings suggest that optimistic expectancies involve particularly strong predictions of reward, causing automatic guidance of attention to reward and great surprise about unexpected punishment. Such great surprise may be counteracted by visual avoidance of the punishing evidence, as revealed by prior evidence, thereby reducing the need to update (over)optimistic reward expectancies.

## Introduction

Most of us are overly optimistic about our future^1,2^. This optimism bias and similar cognitive phenomena such as positive attention bias (preferably attending to positive vs. neutral information in our environment^3^) influence the way we see the world. Despite the fact that both biases are considered as important features of healthy information processing^1,4,5^ and share an association with positive health outcomes^6,7^, optimism bias and positive attention bias have mostly been examined separately.

The combined cognitive biases hypothesis suggests that cognitive biases usually interact and mutually enforce each other and, together, have a greater impact than each bias on its own^8,9^. The combined cognitive biases hypothesis was originally proposed based on findings of a reciprocal relationship between negative self-imagery and biased interpretation in social phobia but has soon been extended to other psychological disorders such as depression (e.g. interacting biases in attention, interpretation, memory, cognitive control)^10^ and anxiety (e.g., expectancy and attention bias interactions)^8^.

Moreover, we^11^ have argued that cognitive bias interactions are not restricted to negative biases that maintain psychological dysfunction. Instead, they may well translate to positivity biases promoting psychological function. Specifically, we proposed a bidirectional interplay, namely (a) optimistic expectancies guide attention to positive information in the environment, and (b) directing attention to positive information enhances optimism bias^11^. Notably, these suggestions are supported by recent empirical findings revealing that induced optimistic expectancies causally influence attention deployment^12^ and that learning to direct one’s attention to positive information through attention bias modification training enhances optimism bias^13^.

If experimentally induced expectancies generate changes in attention, one can assume that biases in expectancies generate biases in attention. Dynamic optimism-attention interactions resulting from such causal influences may then lead to a self-sustaining upward spiral of positivity that protects mental health (e.g. well-being resulting from rewarding experiences and reward-oriented behavior)^6,7^. Even though the identification of the neural mechanisms supporting this dynamic interplay can substantially increase our understanding of how optimism and attention bias maintain over time and support well-being and adaptive reward-oriented behavior, those mechanisms have not yet been experimentally investigated. Neuroimaging studies could, for instance, uncover the key mechanisms that drive the development and maintenance of a positive attention bias by looking into the neural substrates underlying causal influences of reward-related expectancies on attention deployment. One might then go a step further and target divergences and commonalities related to different biases – in health as in psychopathology (e.g. maladaptive cognitions in depression)^11^.

Prior neuroimaging work revealed that attention processes rely on activity in large-scale neural networks^14^. Specifically, the salience network (SN: e.g., anterior insula [AI], dorsal anterior cingulate cortex [DACC]) is crucial for the detection of salient information and initial attention orientation. Furthermore, the SN elicits dynamic shifts between the executive control network (ECN; e.g., dorsolateral prefrontal cortex [dlPFC], posterior parietal cortex [PPC]) and the default mode network (DMN; e.g., medial prefrontal cortex, precuneus). Whereas the ECN is typically *activated* during cognitively demanding tasks (ensuring attention maintenance/modulation of information in working memory), the DMN is typically *deactivated* during cognitively demanding tasks^15,16^.

Studies investigating the impact of expectancies on attention indicate that nodes of the SN and ECN (e.g., AI, dlPFC, PPC, and posterior cingulate cortex [PCC]) are active when spatial attention is shifted to positive information following predictive cues^17–19^. However, the cues in these studies induced expectancies about the spatial location of information (i.e., target will be on the left/right) rather than the information’s valence (i.e., target will be positive/negative). Thus, even though prior findings provide first hints on brain regions underlying the influence of spatial expectancies on attention, little is known about how emotional expectancies (optimism/pessimism) guide attention (see^20^ for investigations of such emotional expectancies on attention in fear).

In a recent publication^11^, we reviewed the neural correlates of both optimism bias and positive attention bias. Moreover, we developed a working model for bidirectional influences between expectancies and attention deployment, emphasizing their dynamic interplay. Specifically, we postulated that expectancy influences on visual attention are mediated as follows: Via top-down mechanisms initiated by an event’s determined saliency (e.g., involving the ACC and other prefrontal areas), optimistic expectancies regarding the accomplishment of a personal goal are supposed to impact executive control (involving the PPC) and direct momentary attention to goal conducive information in the environment (i.e., those pieces of information that are supportive of the initial optimism).

The present fMRI study experimentally tested our model’s predictions and investigated the impact of optimistic and pessimistic expectancies (here defined and operationalized as expectancies for reward and punishment)^21^ on attention deployment and activity in large-scale neural networks. We induced expectancies about future gains and losses and examined their influence on attention to stimuli signaling reward (gain) and punishment (loss). First, in line with earlier findings in the field^12^, we anticipated that both optimistic and pessimistic expectancies guide attention to congruent rather than incongruent information (optimistic expectancies to reward rather than punishment and pessimistic expectancies to punishment rather than reward), whereas processing of incongruent information enhances SN/ECN activity (*congruency hypothesis*)^22^.

Second, and most important, as prior findings suggest that optimism bias is maintained through asymmetric processing of feedback (i.e., greater weight being given to positive than negative feedback)^23^ and we have already shown that such asymmetric processing may be rooted in particularly powerful effects of optimistic expectancies on attention^12^, we hypothesized that optimistic expectancies have a stronger impact on attention and SN/ECN activity than pessimistic ones do (*asymmetry hypothesis*). Notably, our first replication step (*congruency hypothesis*) was intended to identify congruency sensitive SN/ECN brain regions that could then be tested for asymmetric processing, i.e. our main hypothesis. Third, we anticipated a brain-behavior correspondence, namely that asymmetry revealed at the behavioral level is reflected in asymmetric responses at the neural level, specifically in SN/ECN activity (*asymmetry association hypothesis*). Thus, our asymmetry and asymmetry association hypotheses aimed at identifying the neural mechanisms underlying the asymmetric effects displayed at the behavioral level.

## Methods and Materials

### Participants

Fifty healthy participants (19 male, age: *M* = 25.06 years; *SD* = 4.68 years; range = 18–39  years) recruited at the University of Bern took part in this fMRI study. Participants who reported neurological disorders, mental disorders, MRI contraindications, use of psychoactive substances, or left-handedness during a recruitment telephone interview were not included in the study. Furthermore, color blind participants (tested with Ishihara plates)^24^ were excluded. On average, the current sample scored high on the Satisfaction with Life Scale^25^ (*M* = 26.16; *SD* = 6.21, on a scale from 5 to 35) and revealed slight dispositional optimism on the Life Orientation Test-Revised^26^ (*M* = 22.56; *SD* = 3.78, on a scale from 7 to 35), whereas it displayed low trait anxiety on the State Trait Anxiety Inventory^27^ (*M* = 36.02; *SD* = 7.60, on a scale from 20 to 80) and did not meet cut-off scores for depression on the Beck Depression Inventory II^28^ (*M* = 5.08; *SD* = 4.31, on a scale from 0 to 63). All participants had normal/corrected-to-normal vision and were reimbursed with course credit or 25 Swiss francs per hour in addition to 5 Swiss francs (“gain” from the gambling task; see Procedure for details). All participants gave written informed consent and were told that they could end the experiment at any time. All procedures were carried out in accordance to the guidelines of the Declaration of Helsinki and approved by the Swiss Ethics Committee on research involving humans in the canton Bern, Switzerland (https://www.swissethics.ch/index_e.html).

### Procedure

After giving written informed consent, participants read the instructions in which the experiment was described as a gambling task. They were informed to gain 25 Swiss cents in addition to a starting amount of 5 Swiss francs upon seeing a gain target in a visual search array and lose 25 Swiss cents upon seeing a loss target. Participants were told that cues (e.g., “90% gain”) at the beginning of each trial described an average expectancy value of a gain or loss target being subsequently presented in a visual search array. Participants performed six practice trials to become familiar with the task. If they had no questions, they were comfortably positioned in an MRI scanner and the experimental task was performed. For visual stimulation, an LCD projector (PT-L711E, Panasonic, Kadoma, Japan) displayed the stimuli onto a screen in front of the scanner. The screen was viewed through a mirror mounted to the  scanner’s head coil.

**Figure 1:**
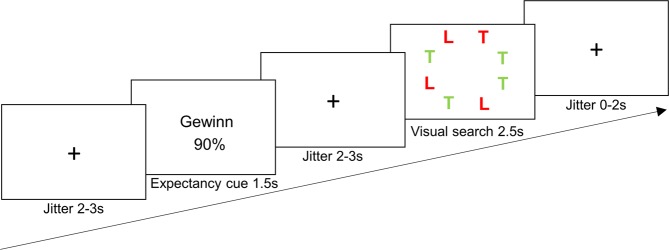
Trial structure. This example trial depicts a gain cue (Gewinn [German word for gain] 90%) preceeding a visual search array containing a gain target (here a red “T”). Participants were  told that cues informed them about an average  likelihood of a gain or loss target being subsequently presented in a visual search array. Participants gained (lost) 25 Swiss cents each time they saw a gain (loss) target in the search array. They were instructed to respond to the target (gain or loss) as quickly and accurately as possible. A similar version of this figure was previously published in a manuscript reporting behavioral data of another study using the same experimental design (Fig. 1)^12^.

Stimuli and experimental task of the current study are identical to Experiment 2 reported in our earlier publication^12^. In each trial, participants were presented with a fixation cross for 2000–3000 ms (jittered presentation) followed by a visual cue presented for 1500 ms (Fig. 1). After the cue, another fixation cross appeared for 2000–3000 ms. Next, a search array consisting of eight stimuli (seven distractors and either a gain or a loss target) was shown for 2500 ms. The participants’ task was to indicate whether the target appeared on the left or right side of the screen by pressing with their right hand´s index or middle finger on a button box connected to a response box outside the scanner (Lumina LP400, Cedrus Corporation, San Pedro, CA). Participants were instructed to react as quickly and accurately as possible. After the detection period had elapsed, another fixation cross was presented for 0–2000 ms before the next trial appeared (total trial duration: 10 s). This task and design was chosen based on prior work investigating expectancy-attention interactions in our lab^12,20,29,30^. E-Prime 2.0 Professional (Psychology Software Tools, Sharpsburg, PA, USA) was used to present stimuli and record our participants’ responses (button presses including reaction times).

A total of 256 trials (128 congruent, 64 incongruent, 64 ambiguous; see Experimental design for details) were presented in random order in four sessions of 64 trials (~11 minutes) with short pauses in between. The frequencies of trials of different kinds (cue-target combinations) were comparable between sessions. In total, participants both gained and lost 32 Swiss francs, leaving them with the starting amount of 5 Swiss francs. Participants were not informed about the progression of their gains and losses during the experiment. After the experiment, participants completed a post-experimental questionnaire as well as additional affect and personality questionnaires that were assessed for a larger project on individual differences associated with optimism bias. Then, participants were debriefed and received their “gain” of 5 Swiss francs (plus their monetary remuneration for taking part in the study, if they did not participate for course credit).

### Experimental design

In the expectancy phase, one of three visual cues was presented to induce optimistic (“gain 90%”), pessimistic (“loss 90%”), or ambiguous (“gain loss 50%” [“loss gain 50%” for half of the participants]) expectancies. These cues indicated the probability that the target in the subsequently presented visual search array would be a gain or a loss target. Because we had to include enough incongruent trials for data analysis, the “gain 90%” (loss 90%”) cue referred to an actual probability of 67% that there would be a gain target (loss target) among seven neutral distractor stimuli in the search array presented afterward. In the remaining cases, a loss target (gain target) was presented. To reduce the participants’ distrust in the cues, they were told that the computer randomly picked a target from a pool of 100 targets (consisting of 90 gain [loss] and 10 loss [gain] targets for 90% gain (loss) cues) and that, therefore, the real expectancy value might differ from the average value displayed by the cues.

In the 50% cue condition, gain and loss targets were equally likely to be the target in the search array. This 50% cue was included as a control condition aiming to induce ambiguous expectancies with maximum uncertainty. However, most participants mentioned before the experiment that they nonetheless believed gain rather than loss targets to appear more frequently than chance level in these ambiguously cued trials (Participants’ answers in a pre-experimental questionnaire to “How often will you gain money when you will be presented with a 50% gain lose (lose gain) cue?” were significantly higher than chance level, whereas there was no significant difference when the same question asked about their expectancy to lose money in ambiguous trials). Similar biases in our “ambiguous condition” have been revealed across different experiments using this design^20^. What is more, the ambiguous cues differed in terms of both valence and predictability from the two other cues, while the optimistic and the pessimistic cues differed in valence only. In addition, verbally stating possibilities of both gains and losses at a time (i.e., gain loss 50% or loss gain 50%), may have provoked confusion in our participants. Consequently, we did not include this ambiguous condition in the current analyses.

Stimuli in the visual search phase consisted of green and red “L”s and green and red “T”s. In each trial, eight red and green “L”s and “T”s were shown on white background on a circle. A single green “L” or a single red “T” served as target stimulus and signaled gain (i.e., reward) and loss (i.e., punishment) of money, respectively. For half of the participants, the green “L” represented a gain and the red “T” represented a loss, whereas for the other half the red “T” represented a gain and the green “L” represented a loss. The target stimulus appeared with equal probability in any of the eight different locations on the circle.

### Behavioral data analysis

Behavioral data were analyzed with IBM SPSS Statistics, Version 24 (International Business Machines Corporation, Armonk, NY, USA). The dependent variable of attention orientation during the visual search task consisted of the participants’ reaction times (RTs) for correct responses (in ms); errors comprised ~7.4% of responses (note that RTs have revealed identical attention patterns as more direct measures of attention orientation such as eye tracking in a previous behavioral study^12^ using the same paradigm. From this, we conclude that RTs provide valid information about attention orientation in this task).

We hypothesized that (1) gain and loss expectancy cues guide attention to congruent rather than incongruent targets (congruency hypothesis). This congruency hypothesis would be supported by faster RTs to expected (i.e., gain targets following gain cues and loss targets following loss cues) than unexpected targets (i.e., loss targets following gain cues and gain targets following loss cues) and was tested with a planned comparison (pairwise t-test) between RTs in the congruent and incongruent conditions.

Furthermore, we hypothesized that (2) this congruency effect would be stronger for optimistic than for pessimistic expectancies (i.e., gain cues guide attention more to gain compared with loss targets than loss cues guide attention to loss compared with gain targets [asymmetry hypothesis]). Therefore, difference scores between RTs of incongruent and congruent conditions were computed for both cues:

$$\begin{array}{c}{{\rm{Diff}}}_{{\rm{GainCue}}}=[{\rm{Gain}}\,{\rm{cue}},\,{\rm{loss}}\,{\rm{target}}]\,\mbox{--}\,[{\rm{Gain}}\,{\rm{cue}},\,{\rm{gain}}\,{\rm{target}}]\\ {{\rm{Diff}}}_{{\rm{LossCue}}}=[{\rm{Loss}}\,{\rm{cue}},\,{\rm{gain}}\,{\rm{target}}]\,\mbox{--}\,[{\rm{Loss}}\,{\rm{cue}},\,{\rm{loss}}\,{\rm{target}}]\end{array}$$

We anticipated larger difference scores for optimistic expectancies than for pessimistic expectancies (Diff_GainCue_ > Diff_LossCue_). This asymmetry hypothesis was tested with a planned comparison (pairwise t-test) between Diff_GainCue_ and Diff_LossCue_. Pairwise *t*-tests were conducted with an α-level of 0.05 (one-tailed) and reported effect sizes are Cohen’s *d*, denoted by *d*.

We also performed all analyses on logarithmic RTs, inverted RTs, or data without outliers (±3 *SD*s from individual average RT). However, the effects observed in the current study were not affected by these data transformations. Therefore, only the results for the original RT data are described.

### FMRI data analysis

All MRI images were acquired using a 3 Tesla Siemens Magnetom Prisma Scanner (Siemens, Erlangen, Germany) with a 64-channel head coil. Volumes were registered using a T_2_*-weighted multi-band echo-planar imaging sequence (multi-band EPI) with 48 slices covering the whole brain (slice thickness = 2 mm; 0.5 mm gap; interleaved slice order; TR = 1000 ms; TE = 30 ms; flip angle = 80°; field of view = 192 × 192 mm; matrix size = 96 × 96; voxel size = 2 × 2 × 2.5 mm; PAT mode GRAPPA; acceleration factor 2; multiband factor = 3). An anatomical scan (MP-RAGE; 1 mm isotropic voxels; TR = 2300 ms; TE = 2.98 ms; flip angle = 9°; matrix size = 256 × 256) was conducted before the functional run to get highly resolved structural information for the normalization procedure.

Statistical Parametric Mapping software (SPM12, Wellcome Department of Cognitive Neurology, London, UK; http://www.fil.ion.ucl.ac.uk/spm) implemented in Matlab R2015b (Mathworks Inc., Sherborn, USA) was used for data analysis. Calculations were performed on UBELIX (https://ubelix.unibe.ch/docs), the high performance computing cluster at the University of Bern. After slice time correction (middle slice acquisition was used as a reference slice), unwarping and spatial realignment (4^th^-degree b-spline interpolation), retrospective noise correction was carried out using the Functional Image Artefact Correction Heuristic Package (FIACH)^31^ implemented in R^32^. Moreover, six principal components of physiological noise regressors were calculated with FIACH. Next, functional data were co-registered to each participant’s anatomical image, normalized to the standard space of the Montreal Neurological Institute (MNI) EPI template to permit group analyses, and spatially smoothed with an isotropic three-dimensional Gaussian filter with a full-width at half maximum (FWHM) of 6 mm.

For statistical analyses, event-related signal changes were modeled separately for each participant, using the general linear model (GLM) as implemented in SPM 12. The following regressors were included in the first-level model separately for each of the four sessions: gain cue, loss cue, ambiguous cue (expectancy phase; duration: 0 s); gain cue–gain target; gain cue–loss target; loss cue–loss target; loss cue–gain target; ambiguous cue–gain target; ambiguous cue–loss target (target phase; duration: 0 s). A parametric modulator describing the modulation of the hemodynamic response in the target phase by the participants’ behavioral responses (standardized RTs) was added for each of the six target phase regressors. Moreover, one regressor for participants’ errors, six movement parameters of the realignment procedure, six physiological noise (e.g. possibly originating from cardiac and respiratory processes)^31^ parameters obtained during noise correction with FIACH (all regressors of no interest), and a constant covariate representing the session-specific mean over scans were implemented in the first-level model. The model included a high-pass filter of 128 s to remove low-frequency drift of the scanner and first-order auto-regressive corrections for auto-correlation between scans.

To test our congruency hypothesis [1], we calculated two contrasts of interest on the individual level and subsequently subjected them to second-level random effects analyses (one-sample t tests), namely: ‘incongruent targets > congruent targets’ (i.e., gain cue-loss target + loss cue-gain target > gain cue-gain target + loss cue-loss target) and ‘congruent targets > incongruent targets’.

To test our asymmetry hypothesis [2], we first calculated separate congruency effects for gain (Diff_GainCue_) and loss cues (Diff_LossCue_) in these congruency-sensitive areas (based on extracted betas for the four experimental conditions in those regions). Identical to our behavioral analyses on asymmetry, we applied a paired samples t-test (α = 0.05, one-tailed) to identify the brain regions that displayed asymmetric congruency effects.

Furthermore, simple regression analyses were conducted to evaluate the association of neural and behavioral (standardized RTs [z across participants]) responses for this asymmetry contrast (Diff_GainCue_ − Diff_LossCue_; asymmetry association hypothesis [3]). For the so-identified regions, we also extracted betas for the four relevant experimental conditions and again calculated Diff_GainCue_ and Diff_LossCue_ that were subsequently compared with paired-samples t-tests. By this, we tested to what degree these areas revealed stronger congruency effects for gain compared to loss cues (i.e., asymmetry hypothesis [2]).

For exploratory whole-brain analyses, we report peak-voxel t statistics for activations that are significant at *p* < 0.05 after whole-brain family-wise error (FWE) random-field theory-based corrections at the voxel level, with an additional cluster-extent threshold of 10 voxels. For regions-of-interest (ROI) analyses, we report peak-voxel statistics using small-volume corrections for reduced search volumes (*p* < 0.05, FDR) and an additional clustering-extent threshold of 10 voxels. Such ROI analyses were performed for the SN and ECN using the small volume correction option of SPM12. Bilateral masks of the SN and ECN (obtained from CAREN)^33^ were used for ROI analyses. The datasets generated and/or analyzed in the current study are available from the corresponding authors on reasonable request.

## Results

### Congruency and asymmetry hypothesis

**Figure 2:**
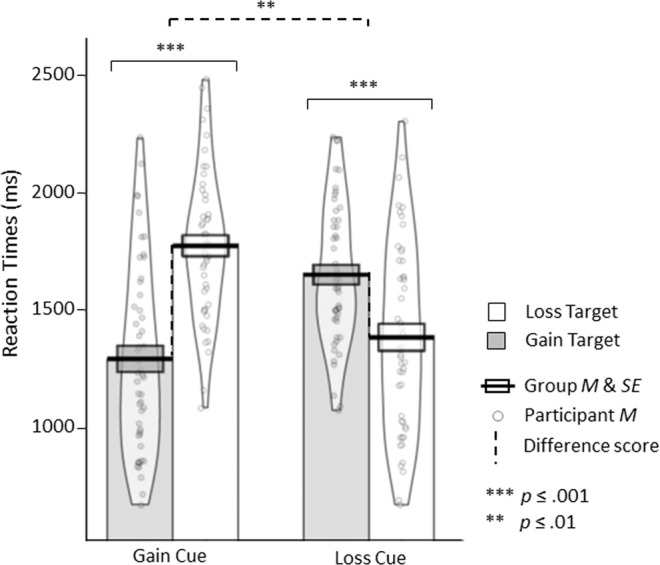
Reaction Times. Bold lines and bands depict mean reaction times and standard errors of all participants (N = 50). Points depict the mean reaction time of each participant. Beans depict smoothed density. Plots were created with the pirate plot function of the Yarrr package Version 0.1.5 (https://CRAN.R-project.org/package=yarrr) in R (R Development Core Team, 2008).

As anticipated, optimistic and pessimistic expectancies guided attention to congruent compared with incongruent information. Participants reacted significantly faster to congruent than incongruent targets, *t*(49) = 9.230, *p* < 0.001, *d* = 1.095 (congruent: *M* = 1341 ms/*SE* = 53 ms, incongruent: *M* = 1716 ms/*SE* = 38 ms; Fig. 2). More important, in line with our asymmetry hypothesis, attention deployment to congruent compared with incongruent targets differed more strongly following optimistic rather than pessimistic expectancies: Optimistic expectancies accelerated behavioral responses to gain compared with loss targets more than pessimistic expectancies accelerated reactions to loss compared with gain targets, *t*(49) = 2.760, *p* = 0.004, *d* = 0.541 (Diff_GainCue_: *M* = 483 ms/*SE* = 52 ms, Diff_LossCue_: *M* = 267 ms/*SE* = 60 ms).

On the neural level, processing of incongruent compared with congruent targets resulted in stronger activation in the bilateral AI (SN), a large bilateral cluster comprising the PPC (superior/inferior parietal lobule [SPL/IPL]; ECN) extending into occipital areas (middle/superior occipital gyrus [MOG/SOG]), a bilateral cluster comprising the supplementary motor area (SMA) extending into the medial frontal gyrus (MeFG) and DACC, bilateral clusters comprising frontal lobe areas (precentral gyrus [PreCG], inferior frontal gyrus [IFG], middle frontal gyrus [MFG], superior frontal gyrus [SFG]), as well as bilateral clusters in the visual cortex (cuneus [CUN]/lingual gyrus [LING]) extending into the PCC (all whole-brain analyses; see Table 1 for a summary of activated clusters).

**Table 1:** Areas displaying differential activation for incongruent versus congruent information during the visual search phase (following both optimistic and pessimistic expectancies).

H	Brain Structure	k	x	y	z	*t*_*max*_	*p*		*M*_*eβ*_				*t*_*As*_	*P*_*As*_
Incongruent > Congruent								Cue	Gain	Gain	Loss	Loss		
Whole-Brain Analysis (FWE corrected)								Target	Gain	Loss	Gain	Loss		
R	AI, IFGor	192	35	25	−3	10.03	<.001		12.74	17.70	17.81	13.34	0.53	.299
B	SPL, IPL, SOG, MOG, ANG (R)	2141	29	−65	45	9.24	<.001		20.59	27.92	28.19	21.71	0.60	.274
B	SMA, MeFG, DACC	597	−4	12	53	8.64	<.001		18.50	25.10	24.66	20.05	1.96	.028
L	PreCG, IFGop, IFGtr, MFG	404	−44	6	35	8.29	<.001		16.35	23.36	22.58	17.96	1.73	.045
L	AI	88	−32	21	−5	8.09	<.001		7.04	10.88	11.00	8.27	1.50	.069
R	MFG, SFG	94	31	2	58	7.28	<.001		10.10	14.24	13.48	11.23	2.14	.018
R	PCC, CUN, LING	66	11	−65	10	6.87	.001		14.34	17.81	17.64	14.37	0.20	.420
L	MFG, SFG	63	−24	0	60	6.84	.001		16.52	21.45	20.25	17.58	2.54	.007
R	IFGop, IFGtr, MFG	86	49	25	30	6.75	.001		3.50	9.10	10.19	4.28	−0.25	—
L	CUN, PCC, LING	51	−14	−71	8	6.53	.003		13.92	18.12	17.50	14.64	1.16	.125
R	IFGop, PreCG	22	37	6	30	6.32	.006		12.14	16.74	16.06	13.49	2.21	.016
R	IFGop, PreCG, MFG	32	49	10	33	6.27	.007		17.31	22.85	23.03	18.52	0.75	.230
L	CUN, LING	18	−2	−75	5	6.25	.008		7.74	11.52	11.33	8.46	0.90	.185
R	IOG	13	31	−85	−5	6.22	.009		10.24	12.73	13.10	10.42	−0.16	—
**Region-of-Interest Analysis (FDR corrected)**														
*Salience Network*														
R	AI, IFGtr	124	37	23	−3	9.16	<.001		11.47	15.46	16.22	12.05	−0.19	—
R	SMA, DACC, MeFG, SFG	129	7	8	55	7.18	<.001		17.16	22.96	22.10	18.24	2.12	.019
L	DACC, SMA, MeFG	82	−6	12	50	8.24	< .001		16.77	20.82	19.99	17.61	1.68	.050
L	AI, IFGtr	106	−36	19	−5	5.80	<.001		5.41	8.51	8.68	6.15	0.77	.223
*Executive Control Network*														
R	AI, IFGor	68	35	25	−3	10.03	<.001		12.74	17.70	17.81	13.34	0.53	.299
R	SPL, IPL, MOG, PostCG	613	27	−63	45	8.88	<.001		20.77	27.31	27.59	21.92	0.70	.244
L	SPL, IPL, MOG	751	−28	−57	53	8.16	<.001		30.26	37.87	37.09	32.00	2.03	.024
L	AI, IFGor	70	−32	21	−5	8.09	<.001		7.04	10.88	11.00	8.27	1.50	.069
L	SMA, SFG	56	−4	10	55	7.93	<.001		18.09	24.34	23.97	19.73	2.07	.022
L	SMA, MeFG, DACC	72	−6	19	48	7.83	<.001		8.25	12.64	12.93	9.22	0.78	.220
L	MFG, IFGtr, IFGop, PreCG, SFG	608	−46	6	33	7.49	<.001		17.03	23.52	22.84	18.18	1.39	.085
R	SMA, MeFG, DACC	71	5	19	48	7.07	<.001		12.07	16.36	16.14	13.26	1.47	.074
R	MFG, IFGtr	197	49	27	28	6.68	.001		4.79	9.81	10.70	5.51	−0.13	—
R	SFG, MFG	49	25	6	58	6.37	.001		10.13	13.43	13.82	10.69	0.18	.428
R	IFGop, PreCG	69	37	6	30	6.32	.001		12.14	16.74	16.06	13.49	2.21	.016
R	MFG	62	37	4	60	5.67	.007		3.178	6.69	7.82	4.44	0.13	.447
L	ITG	25	−48	−55	−15	4.93	.007		2.71	5.24	4.76	2.99	1.08	.143
L	SFG, MFG	15	−26	47	18	4.70	.020		3.48	6.17	6.57	4.58	0.76	.227
L	MFG	80	−40	51	3	4.60	.031		−3.26	−0.28	1.11	−2.73	−0.57	—
R	IFGop	22	49	14	13	4.35	.049		1.53	3.60	3.71	1.72	0.11	.455
**Congruent > Incongruent**														
**Whole-Brain Analysis (FWE corrected)**														
B	MOFC	110	−2	41	−13	7.07	<.001		−5.40	−9.32	−8.17	−6.17	1.87	.034
L	IPL, PostCG	38	−65	−30	30	6.66	.002		−9.09	−13.80	−12.65	−9.83	1.53	.067
R	IPL, PostCG	37	64	−24	20	6.64	.002		−9.30	−14.52	−14.41	−10.94	1.54	.065
**Region-of-Interest Analysis (FDR corrected)**														
*Salience Network*														
L	IPL, PostCG	205	−65	−30	30	6.66	.003		−9.09	−13.80	−12.65	−9.83	1.53	.067
R	IPL, PostCG	220	60	−20	18	5.82	.033		−2.32	−6.97	−6.05	−3.90	1.87	.034

Note. All coordinates (x, y, z) of peak voxel activation are given in Montreal Neurological Institute (MNI) space. H = hemisphere;  L = left, R = right, B = bilateral; *k* = cluster size in number of voxels (voxel size = 2 ×2 ×2.5 mm); *M*_*eβ*_ = mean estimated beta, *t*_*As*_ and *p*_*As*_ refer to t and p values related to our asymmetry hypothesis (see Methods and Materials for details). *p*_*As*_ is not specified for those t values that are incongruent with our hypothesis. Note that some results are listed for both SN and ECN (e.g. AI) as the network masks we used slightly overlapped. This is due to the fact that the SN and ECN networks are not identical across the different atlases at the basis of CAREN^33^. Such overlap between SN and ECN masks might further relate to interactions between SN and ECN. For exploratory whole-brain analyses, a clustering threshold of *p* < 0.05, whole-brain FWE corrected, and an additional cluster-extent threshold of 10 voxels was used; ROI analyses involved FDR correction, with an additional cluster-extent of 10 voxels. *AI* = Anterior Insula, *ANG* = Angular Gyrus, *CUN* = Cuneus, *DACC* = Dorsal Anterior Cingulate Cortex, *IFGop* = Inferior Frontal Gyrus - pars opercularis, *IFGor* = Inferior Frontal Gyrus – pars orbitalis, *IFGtr* = Inferior Frontal Gyrus – pars triangularis, *IOC* = Inferior Occipital Gyrus, *IPL* = Inferior Parietal Lobule, *ITG* = Inferior Temporal Gyrus, *LING* = Lingual Gyrus, *MeFG* = Medial Frontal Gyrus, *MFG* = Middle Frontal Gyrus, *MOFC* = Medial Orbitofrontal Cortex, *MOG* = Middle Occipital Gyrus, *PCC* = Posterior Cingulate Cortex, *PreCG* = Precentral Gyrus, *PostCG* = Postcentral Gyrus, *SFG* = Superior Frontal Gyrus, *SMA* = Supplementary Motor Area, *SOG* = Superior Occipital Gyrus, *SPL* = Superior Parietal Lobule.

**Figure 3:**
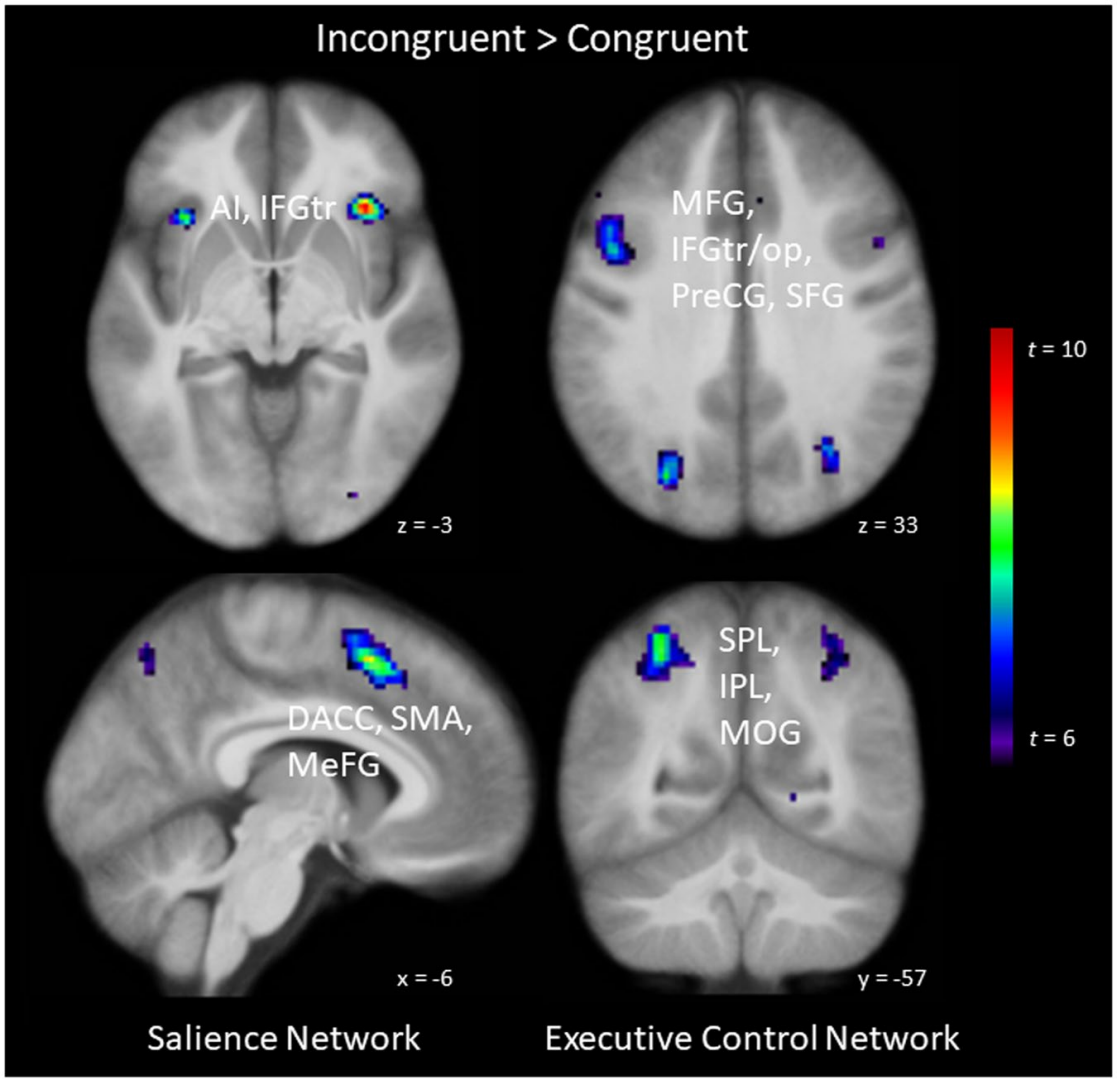
Brain areas displaying differential activation on viewing incongruent compared with congruent information during the visual search phase. Processing incongruent information elicits stronger activation in nodes of the salience network (anterior insula [AI], inferior frontal gyrus – pars triangularis [IFGtr], dorsal anterior cingulate cortex [DACC], supplementary motor area [SMA], medial frontal gyrus [MeFG]) and of the executive control network (middle frontal gyrus [MFG], IFGtr, inferior frontal gyrus – pars opercularis [IFGop], precentral gyrus [PreCG], superior frontal gyrus [SFG], superior parietal lobule [SPL], inferior parietal lobule [IPL], middle occipital gyrus [MOG]) than processing congruent information following both optimistic and pessimistic expectancies. Statistical parametric maps are thresholded at *p* < 0.05, whole-brain corrected.

Consistent with this picture, ROI analyses for the SN revealed that processing of incongruent rather than congruent targets resulted in stronger activation in the bilateral AI (extending into the IFG) and DACC (extending into SMA and MeFG). ROI analyses for the ECN revealed that processing of incongruent rather than congruent targets additionally resulted in stronger activation in a bilateral cluster comprising the PPC (SPL/IPL) and bilateral clusters comprising the IFG, MFG, and SFG (see Fig. 3 for a visualization of activations). Importantly, contrasts performed on the extracted betas of those regions showed that the pattern of activation in almost all above described regions accords with the asymmetry we predicted based on our behavioral data: Processing incongruent vs. congruent targets led to larger activation differences in the SN and ECN following gain compared to loss expectancies (revealed by both whole-brain and ROI analyses; see Table 1). The direction of the effect was correctly predicted in 12 out of 14 contrasts performed at the whole brain level (*p* = 0.0056, according to the binomial distribution; 5 of those contrasts reaching significance), and 17 out of 20 contrasts performed for our ROI analyses (*p* = 0.0011; 5 contrasts reaching significance).

In contrast, processing of congruent compared with incongruent targets resulted in stronger activation in a bilateral cluster in the medial orbitofrontal cortex (MOFG) and a bilateral cluster comprising of IPL and postcentral gyrus (PostCG) (whole-brain corrected). Correspondingly, ROI analyses for the SN revealed that processing of congruent rather than incongruent targets resulted in stronger activation in a bilateral cluster comprising of IPL and PostCG. Again, analyses of the extracted betas from these areas revealed an asymmetric pattern of activation (i.e. larger activation differences when processing incongruent vs. congruent targets following gain compared to loss expectancies). The direction of the effect was correctly predicted in 5 out of 5 contrasts performed, with 2 of these contrasts achieving significance.

We also performed separate congruency contrasts for optimistic and pessimistic cues. Corresponding results regarding the neural activation during processing of incongruent vs. congruent information can be found in Supplementary Table 1.

### Asymmetry association (and asymmetry) hypothesis

Participants’ RTs reflecting asymmetric attention deployment following optimistic and pessimistic expectancies was predicted by *enhanced* activation in bilateral clusters in frontal lobe areas (PreCG, IFG), bilateral SMA (extending to SFG and MeFG), and clusters comprising of the left PPC (SPL, IPL; nodes of the ECN) as well as MFG and SOG when processing incongruent information following optimistic vs. pessimistic expectancies (whole-brain analyses).

**Figure 4:**
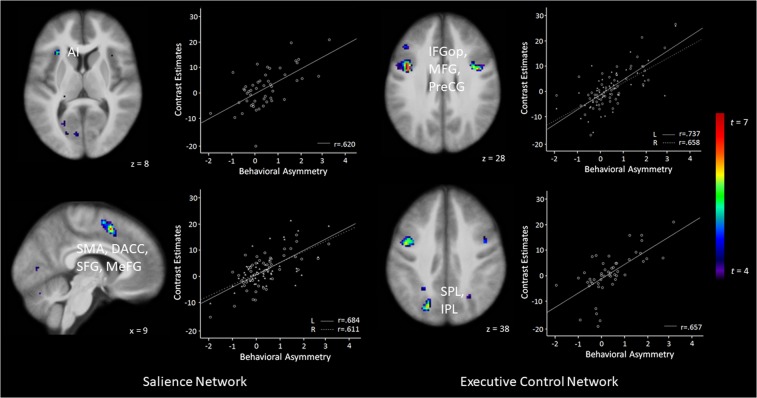
Brain areas displaying differential activity to unexpected punishment (vs. expected reward) following optimistic expectancies compared with unexpected reward (vs. expected punishment) following pessimistic expectancies in the visual search phase predicting asymmetric attention deployment following optimistic compared with pessimistic expectancies indicated by RTs (Diff_GainCue_ > Diff_LossCue_). Participants demonstrating strongest asymmetric attention deployment following optimistic vs. pessimistic expectancies (indicated by RTs) also show the strongest activity in nodes of the salience network (anterior insula [AI], supplementary motor area [SMA], dorsal anterior cingulate cortex [DACC], superior fronal gyrus [SFG], medial frontal gyrus [MeFG]) and the executive control network (inferior frontal gyrus – pars opercularis [IFGop], middle frontal gyrus [MFG], precentral gyrus [PreCG], superior parietal lobule [SPL], inferior parietal lobule [IPL]) when processing unexpected vs. expected information following optimistic vs. pessimistic expectancies. Statistical parametric maps are thresholded at *p* < 0.001, uncorrected, for visualization purposes. See Table 2 for corrected inferential statistics. L = Left, R = Right.

Moreover, ROI analyses for the SN revealed that behavioral responses reflecting asymmetric attention deployment were predicted by *enhanced* activation in the left AI, a bilateral cluster comprising of DACC, SMA and MeFG as well as the right SFG when reorienting attention to incongruent information following optimistic vs. pessimistic expectancies (see Fig. 4 for visualization). ROI analyses for the ECN further demonstrated that behavioral responses reflecting asymmetric attention deployment were predicted by *enhanced* activation in a bilateral cluster comprising of the IFG and PreCG as well as clusters in the left SMA and SFG, left IPL and SPL, and left SFG and MeFG. Analyses performed on the extracted betas once more revealed that the pattern of activation in almost all described regions shows the predicted asymmetry, i.e. processing incongruent vs. congruent targets led to larger activation differences in the SN and ECN following gain compared to loss expectancies (revealed by both whole-brain and ROI analyses; see Table 2). The direction of the effect was correctly predicted in 7 out of 7 contrasts performed at the whole brain level (*p* = 0.0078; 2 contrasts reaching significance), and 8 out of 8 contrasts performed for our ROI analyses (*p* = 0.0039; 3 contrasts reaching significance).

**Table 2:** Areas displaying differential activity to unexpected punishment (vs. expected reward) following optimistic expectancies compared with unexpected reward (vs. expected punishment) following pessimistic expectancies in the visual search phase predicting asymmetric attention deployment following optimistic compared with pessimistic expectancies indicated by RTs (Diff_GainCue_ > Diff_LossCue_). Scatterplots displaying the relation between behavioral and neural asymmetry for each area can be found in the supplementary materials (Supplementary Figure 1).

H	Brain Structure	k	x	y	z	*t*_*max*_	*p*		*M*_*eβ*_				*t*_*As*_	*P*_*As*_
Positive Correlation								Cue	Gain	Gain	Loss	Loss		
Whole-Brain Analysis (FWE corrected)								Target	Gain	Loss	Gain	Loss		
L	IFGop, PreCG	72	−40	4	28	7.56	<.001		11.75	16.65	16.59	13.39	1.57	.062
L	PreCG	32	−42	−6	45	7.27	<.001		10.34	12.91	13.03	11.48	1.26	.106
B	SMA, SFG, MeFG	61	0	10	60	6.83	.001		11.78	17.16	16.59	13.55	1.99	.026
L	SPL, IPL	18	−26	−55	45	6.66	.002		17.95	22.82	22.72	19.06	1.24	.110
L	SPL, SOG	10	−20	−71	40	6.59	.002		18.12	22.94	22.17	19.02	1.69	.049
R	IFGop, PreCG	12	43	2	30	6.45	.004		12.56	15.73	15.96	13.30	0.54	.297
L	PreCG, MFG	16	−30	−6	55	6.2	.010		16.24	20.38	19.86	17.15	1.63	.055
**Region-of-Interest Analysis (FDR corrected)**														
*Salience Network*														
L	SMA, DACC, MeFG	41	−6	8	53	6.49	.002		18.39	23.36	23.07	19.39	1.50	.070
L	AI	38	−30	23	8	5.47	.018		13.63	17.41	16.84	14.63	1.48	.072
R	SMA, DACC, SFG, MeFG	63	9	10	53	5.35	.027		13.02	16.10	15.28	13.76	1.79	.040
*Executive Control Network*														
L	IFGop, MFG, PreCG	187	−40	4	28	7.56	.001		11.75	16.65	16.59	13.39	1.57	.062
L	SMA, SFG	25	−4	8	55	6.23	.004		20.42	26.67	26.31	21.96	2.06	.022
R	IFGop, PreCG	41	41	4	30	6.05	.006		15.30	19.50	19.62	16.43	0.98	.166
L	SPL, IPL	235	−22	−71	38	6.04	.006		18.63	23.61	23.04	19.36	1.15	.128
L	SFG, MeFG	38	−22	−2	58	5.55	.018		16.78	20.71	20.09	17.81	2.20	.016
**Negative Correlation**														
**Whole-Brain Analysis (FWE corrected)**														
L	IPL, STG	48	−61	−57	25	6.39	.005		−10.63	−13.06	−10.37	−11.67	−2.55	.007
**Region-of-Interest Analysis (FDR corrected)**														
*Salience Network*														
R	IPL	59	−59	−39	40	5.91	.018		−16.78	−20.00	−18.13	−17.31	1.59	.060
*Executive Control Network*														
L	IPL	36	−57	−39	43	5.77	.021		−13.88	−16.26	−13.90	−14.21	1.88	.033
R	MFG	47	43	21	48	5.50	.030		−10.08	−9.36	−7.34	−10.33	−1.56	—
R	MTG	14	64	−53	8	5.45	.030		−6.98	−9.73	−8.37	−7.81	1.72	.046

Note. All coordinates (x, y, z) of peak voxel activation are given in Montreal Neurological Institute (MNI) space. H = hemisphere; L = left, R = right, B = bilateral; *k* = cluster size in number of voxels (voxel size = 2 ×2 ×2.5 mm); *M*_*eβ*_ = mean estimated beta, *t*_*As*_ and *p*_*As*_ refer to t and p values related to our asymmetry hypothesis (see Methods and Materials for details). *p*_*As*_ is not specified for those t values that are incongruent with our hypothesis. For exploratory whole-brain analyses, a clustering threshold of *p* < 0.05, whole-brain FWE corrected, and an additional cluster-extent threshold of 10 voxels was used; ROI analyses involved FDR correction, with an additional cluster-extent of 10 voxels. *AI* = Anterior Insula, *DACC* = Dorsal Anterior Cingulate Cortex, *IFGop* = Inferior Frontal Gyrus - pars opercularis, *IPL* = Inferior Parietal Lobule, *MeFG* = Medial Frontal Gyrus, *MFG* = Middle Frontal Gyrus, *MTG* = Middle Temporal Gyrus, *PreCG* = Precentral Gyrus, *SFG* = Superior Frontal Gyrus, STG = Superior Temporal Gyrus, *SOG* = Superior Occipital Gyrus, *SMA* = Supplementary Motor Area, *SPL* = Superior Parietal Lobule, *STG* = Superior Temporal Gyrus.

Furthermore, behavioral responses reflecting asymmetric attention deployment were predicted by *reduced* activation in a cluster comprising the left IPL and SPL when processing incongruent information following optimistic vs. pessimistic expectancies (whole-brain corrected). ROI analyses for the SN and ECN further revealed that behavioral responses reflecting asymmetric attention deployment were predicted by *reduced* activation in bilateral IPL as well as the right MFG and middle temporal gyrus (MTL). Notably, findings based on the analysis of the extracted betas in these regions are overall consistent with our asymmetry hypothesis (revealed by both whole-brain and ROI analyses; see Table 2). The direction of the effect was correctly predicted in 1 out of 1 contrasts performed at the whole brain level (1 contrast reaching significance), and 3 out of 4 contrasts performed for our ROI analyses (2 contrasts reaching significance).

## Discussion

The current study replicates prior behavioral findings showing that optimistic (and pessimistic) expectancies causally influence attention deployment (i.e., accelerate behavioral responding to congruent compared with incongruent information)^12,34^. In addition, the current findings reveal the importance of large-scale neural networks underlying such expectancy effects on attention. Specifically, induced optimistic and pessimistic expectancies guide attention to congruent information and result in enhanced activity in SN (AI, DACC) and ECN (PPC) nodes during processing of incongruent information. Whereas the SN underlies the detection of salient (here incongruent) information, the ECN underlies further processing (e.g., cognitive control and modulation) of such salient information^16,35^. These findings suggest that optimistic and pessimistic expectancies create a mental template that facilitates the detection of expected information and allows the brain to devote relatively little resources to expected information (as proposed by predictive coding theory)^22,36,37^. By contrast, unexpected information elicits conflict processing and requires additional cognitive and neural resources (as suggested by cognitive control theory)^38^.

Our results are in line with recent findings indicating that attention deployment to neutral information and associated brain activity in frontal and parietal areas is modulated by prior expectancies^20^. Here, we additionally found strong AI and DACC activity during processing of unexpected information following optimistic and pessimistic expectancies, conforming to theoretical considerations on the neurophysiological basis of optimism-attention interactions^11^. The AI plays a crucial role in a variety of tasks concerning subjective awareness of positive and negative feelings^16,39,40^ and may, therefore, be particularly important for salience detection of unexpected reward/punishment compared with unexpected neutral information.

Most important, the observed effects of expectancies on attention show a clear asymmetry: Both the reported behavioral and neural effects were stronger for optimistic than for pessimistic expectancies. Optimistic expectancies guided attention particularly fast to reward (indicated by accelerated behavioral responses) and resulted in strong SN (particularly so in DACC/SMA) and ECN activity (e.g., IPL/SPL and IFGop/PreCG) during the processing of unexpected punishment. Asymmetry was even more pronounced when taking individual differences into account. Participants who demonstrated the strongest asymmetry in behavioral responding following optimistic and pessimistic expectancies also demonstrated the strongest asymmetry in neural responding in the SN (e.g., AI and SMA/DACC) and the ECN (e.g., IPL/SPL and IFGop/PreCG). Note that our neural model incorporated RTs as parametric modulators, thereby ensuring that the observed effects are not due to motor responses. Thus, people whose reactions to reward were particularly fast following optimistic expectancies also displayed particularly strong SN and ECN activation during the processing of unexpected punishment.

In addition, we found behavioral asymmetry scores to correlate negatively with neural activity in several areas (e.g., in IPL, MFG, and MTG) that were characterized by stronger *de*activations to incongruent compared with congruent stimuli for optimistic vs. pessimistic expectancies. While we had not predicted these deactivations in the first place, the observation of greater congruency effects for gain compared with loss cues is consistent with our idea of optimistic expectancies being more powerful than pessimistic expectancies.

Asymmetric attention deployment and neural processing following optimistic and pessimistic expectancies in the current study complement prior findings on asymmetric information processing related to optimism bias^23,41^. People selectively update future expectancies following positive feedback but not following negative feedback – a key process maintaining optimism bias^1^. Selective attention to reward and strong neural processing of unexpected punishment following optimistic expectancies as revealed by our study may constitute important mechanisms underlying this updating asymmetry. Whereas our behavioral data reveal the exceptional power of optimistic expectancies in guiding visual attention to reward, our neural data suggest that unexpected punishment comprises strong expectancy violation^42^ and is especially surprising/salient when people are optimistic (as indexed by enhanced SN activity). Even though unexpected punishment following optimistic expectancies is thoroughly processed in the brain (in line with the idea that prediction errors enhance neural processing)^22^, such processing might take time. Accordingly, those kinds of expectancy violations might be so surprising that they cannot easily be translated into behavior^43^. Instead, participants may determine that such violations are exceptional and keep with their initial (over)optimistic expectancies.

Our findings can be integrated with research on set shifting^44^. Reacting to a stimulus other than the one expected requires participants to actively shift their attention away from the expected information, a process that has been shown to be related to activity in orbitofrontal and anterior cingulate cortices of the brain^44–46^. The current data suggests that it may be more difficult for participants to shift their attention away from reward (i.e. gain expectancies) to punishment (i.e. loss targets) rather than away from punishment (i.e. loss expectancies) to reward (i.e. gain targets). Such asymmetric attention shifting could be a possible mechanism ensuring preferred processing of reward-related information, thereby contributing to biases at various levels (e.g. attention and expectancies).

Supporting these initial effects, later, more controlled attention maintenance on reward compared with punishment (as observed in our earlier publication^12^) can then act as a form of emotion regulation^47–49^ reducing surprising punishing information´s salience^50^. Correspondingly, ECN activity has been suggested to underlie emotion regulation via attention processes and manipulate information in working memory for sustained goal-relevant and adaptive processing^15,51,52^. ECN nodes observed to be active in the current study may, therefore, be involved in the down-regulation of punishing information’s salience following optimistic expectancies. Together, such regulative actions may stabilize optimism over time.

The current data in combination with our earlier findings^12^ imply that optimistic expectancies are particularly robust even in the presence of punishing information. Such asymmetric attention deployment and neural processing following optimistic and pessimistic expectancies may, in turn, explain why people selectively update future expectancies into an optimistic, not a pessimistic direction following feedback^53^: Strong reward predictions automatically guide and maintain attention on reward, thereby reducing disproving punishing information’s salience and prioritizing rewarding information when updating expectancies.

The neural mechanisms underlying the influence of optimistic expectancies on attention uncovered by the current study provide a more nuanced view on how optimism is maintained. Whereas purely behavioral observations may easily lead to the conclusion that asymmetric updating of expectancies after receiving positive feedback^23,41^ is rooted in more thorough processing of reward following overly pessimistic expectancies^12^, our neural data imply that the opposite might actually be true. Disproving punishing information following optimistic expectancies is intensely processed by the brain. Such strong processing of salient negative information following optimistic expectancies can be crucial to differentiate between relevant (e.g., life-threatening situations in which erroneous optimistic expectancies need to be overridden)^20,54^ and irrelevant (e.g., achievement situations in which punishing information diminishes motivation)^11^ negative information. In the latter scenario, more controlled attention processes may serve the purpose to reduce unexpected punishment´s salience, thereby maintaining an optimistic outlook^47,48,50^.

Unfortunately, we did not directly measure attention maintenance in the present study, which limits the conclusions on these processes. However, we have previously shown that people maintain attention on rewarding information following optimistic expectancies in two independent studies using the identical experimental paradigm^12^. Because findings on attention orientation in the current study replicated our previous work, one can strongly assume that the same would be true for attention maintenance. Nevertheless, it is important that future research simultaneously assesses both attention orientation and maintenance (e.g., through eye tracking) in addition to neural and somatovisceral responses to provide an elaborative view on asymmetric attention deployment following optimistic and pessimistic expectancies.

Somewhat unexpectedly, we found a small number of areas (within IPL, MFG, and MTG; mostly located in the ECN), in which asymmetric neural activity was negatively correlated with the asymmetry in our participant’s behavior. While the asymmetry revealed in these areas was consistent with our asymmetry hypothesis, the negative correlation can be explained by the fact that those areas were characterized by deactivations rather than activations. It remains to be clarified, why these deactivations arose in the first place. One possibility is that they are actually related to differences in attention maintenance between the experimental conditions (e.g., controlled withdrawal of attention from unexpected punishment and controlled maintenance of attention to reward in case of prior optimistic expectancies). The function of other areas involved when expectancies guide attention (i.e., additional regions that were identified in our whole-brain analyses) needs to be determined as well. Because we had no clear a-priori hypotheses regarding these areas, we refrained from (over)interpreting their respective involvements.

Furthermore, the terms optimism and pessimism often refer to distal rather than immediate outcomes in the literature (e.g. whether one will live past a certain age or have a heart attack in the future)^2^, whereas we operationalized optimism and pessimism with regards to immediate monetary rewards or losses in the current study. Importantly, recent findings revealed quantitative but not qualitative differences between (optimistic) expectancies measured at different time points^55^, suggesting that the effects we observed in the current research might translate to distal outcomes. Future research should aim to replicate the current findings with stimuli signaling reward/punishment after longer time intervals to explore whether the findings of the current study do indeed translate to expectancies about more distal outcomes.

Another criticism of the current study may relate to the rather short interstimulus intervals used, which makes it harder to distinguish the BOLD signal related to the different events. However, we argue that the use of a multi-band EPI sequence with high temporal resolution (i.e. a short TR of 1000 ms) allowed us to reliably distinguish between cues and targets despite their rapid succession^20^. The current design was based on another recently published fMRI study from our lab using similar interstimulus intervals^20^, which are - though short - not uncommon in the literature^56^.

In conclusion, the present findings further expand our understanding of the mechanisms underlying dynamic optimism-attention interactions. Our data indicate that even though optimistic expectancies guide attention to reward, unexpected punishment following those expectancies is profoundly processed in the brain. These results emphasize the importance of additional mechanisms involved in information processing following optimistic expectancies (i.e., controlled attention maintenance on reward to reduce the salience of unexpected punishment). This can give first hints on how unfavorable interacting cognitive biases (e.g., reflected in a failure to downregulate salience of punishment, thereby initiating a downward spiral of negativity) may be interrupted in psychopathology. Thus, the present findings have brought us a significant step further to unraveling how an optimistic outlook about our future influences the way we process the information around us and could further stimulate future research on how such processing in turn may help us stay optimistic^57^.

## Supplementary information

Supplementary materials.

## Data Availability

The datasets generated and/or analyzed in the current study are available from the corresponding authors upon reasonable request.
