# Supplementary material for: The effect of optimistic expectancies on attention bias: Neural and behavioral correlates
Source: Sci Rep. 2020 Apr 16;10:6495. doi: 10.1038/s41598-020-61440-1 (PMC7162893; doi:10.1038/s41598-020-61440-1)
Supplement: Supplementary file 1 — Supplementary materials. [file 41598_2020_61440_MOESM1_ESM.pdf]

Supplementary materials

The effect of optimistic expectancies on attention bias: Neural and behavioral correlates

Singh, L., Schuepbach, L., Moser, D. A., Wiest, R., Hermans, E. J., & Aue, T.

Supplementary Table 1. Areas displaying differential activation for incongruent versus congruent information during the visual search phase following optimistic and pessimistic expectancies separately.

H	Brain Region	*k*	x	y	z	T_{max}	p_{FWE}
	**Optimistic expectancies: Gain Target > Loss Target**						
	Whole-Brain Analysis						
R	SMG, STG	45	64	-22	25	6.6	.002
L	MOFC	20	-2	45	-10	6.24	.008
	**Optimistic expectancies: Loss Target > Gain Target**						
	Whole-Brain Analysis						
R	SMA, MeFG, MCA	426	-4	14	48	8.78	<.001
R	INS	102	33	25	-3	8.22	<.001
L	INS	38	-34	21	-5	8.09	<.001
L	SOG, MOG, SPL, IPL, PCU	625	-24	-73	38	7.82	<.001
L	PreCG, IFGpo, IFGpt	360	-38	0	50	7.78	<.001
R	SOG, MOG, SPL, IPL, PCU, ANG	471	27	-63	40	7.66	<.001
R	MFG, SFG	68	33	2	58	7.45	<.001
L	SFG, MFG, PreCG	112	-24	0	63	7.41	<.001
R	SMG	14	41	-39	43	6.87	.001
R	IFGpo, PreCG	11	37	6	30	6.34	.006
	**Pessimistic expectancies: Gain Target > Loss Target**						
	Whole-Brain Analysis						
R	MeFG	55	5	35	53	7.46	<.001

L	IPL, SPL, SOG, MOG	295	-36	-51	43	7.28	<.001
R	SPL, SOG, ANG	165	25	-69	53	7.15	<.001
R	INS	36	35	23	-3	6.84	.001
R	IFGpt	14	49	25	30	6.3	.006
**Pessimistic expectancies: Loss Target > Gain Target**							
Whole-Brain Analysis							
no significant results							

Note. All coordinates (x, y, z) of peak voxel activation are given in Montreal Neurological Institute (MNI) space; *k* = cluster size in number of voxels (voxel size = 2 x 2 x 2.5 mm); L = left, R = right, B = bilateral; for exploratory whole-brain analyses, a clustering threshold of *p* <.05, whole-brain FWE corrected, and an additional cluster-extent threshold of 10 voxels was used; *ANG* = Angular Gyrus, *CAL* = Calcarine Sulcus, *IFGpo* = Inferior Frontal Gyrus - pars opercularis, *IFGpt* = Inferior Frontal Gyrus – pars triangularis, *INS* = Insula, *IPL* = Inferior Parietal Lobule, *LING* = Lingual Gyrus, *MCA* = Midcingulate Area, *MeFG* = Medial Frontal Gyrus, *MFG* = Middle Frontal Gyrus, *MOFC* = Medial Orbitofrontal Cortex, *MOG* = Middle Occipital Gyrus, *PCU* = Precuneus, *PreCG* = Precentral Gyrus , *SFG* = Superior Frontal Gyrus, *SOG* = Superior Occipital Gyrus, *SMA* = Supplementary Motor Area, *SMG* = Supramarginal Gyrus, *SPL* = Superior Parietal Lobule, *STG* = Superior Temporal Gyrus.

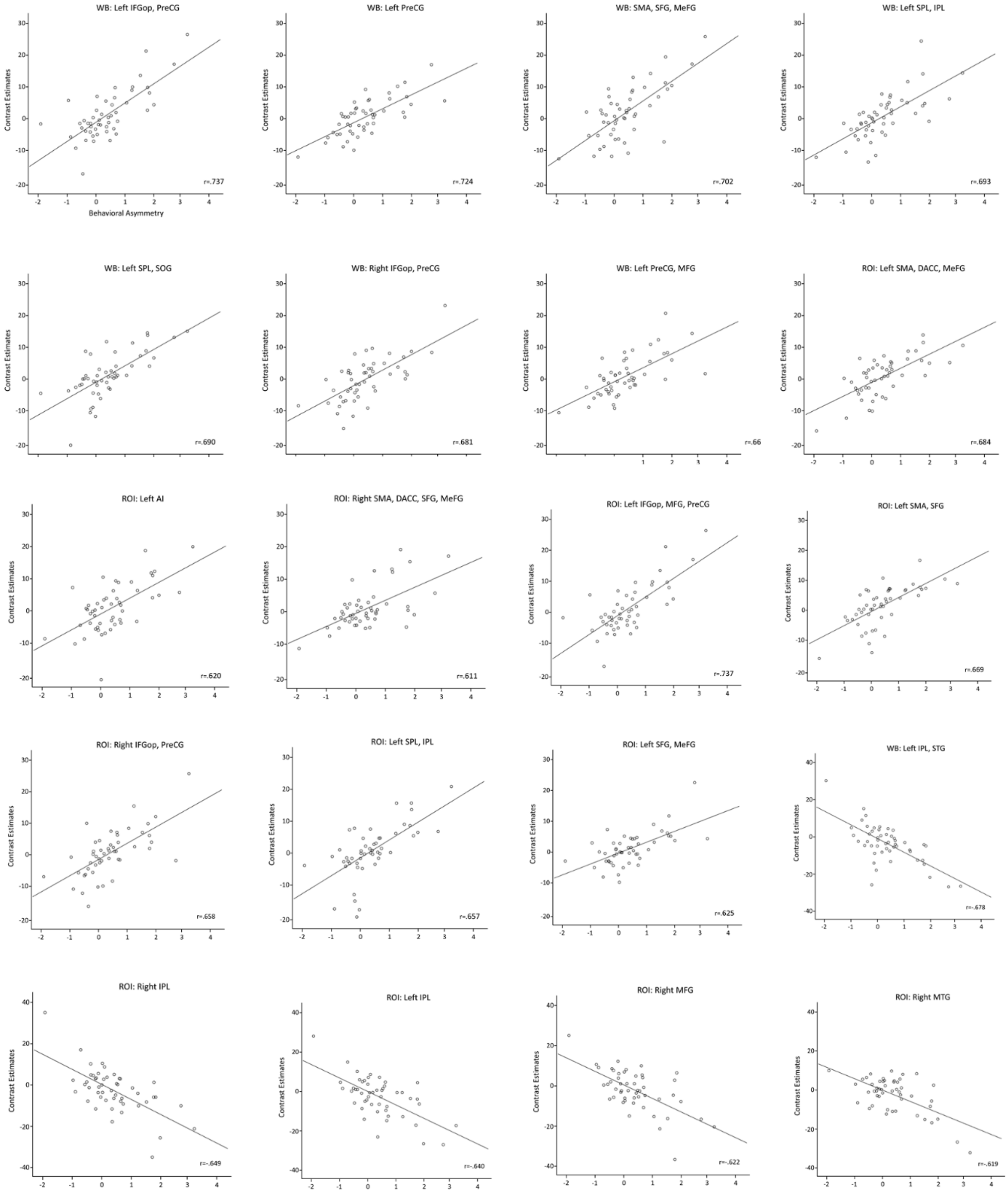

Supplementary Figure 1. Scatterplots displaying the relation between neural and behavioral asymmetry. Behavioral asymmetry: (DiffGainCue > DiffLossCue) in reaction times. Neural asymmetry score: (DiffGainCue > DiffLossCue) in neural activity related to the visual search phase in areas specified in Table 2.
